# 

**Published:** 1908-01-18

**Authors:** 


### THE GRADUATES' MEDICAL SCHOOLS.

### Diary for the week January 20 to 25.

**THE THROAT HOSPITAL, Golden Square, W.**

**Lectures at 5.30 p.m.**

**Jan. 20,** Mr. C. A. Parker, **Affections of the Auricle and External Auditory Canal.**

**Jan. 23,** Dr. H. W. F. Powell, **Acute Inflammatory Affections—Simple.**

**THE POST-GRADUATE COLLEGE, West London Hospital, Hammersmith, W.**

**Lectures: At noon.**

**Jan. 20,** Dr. Low, **Pathological Demonstration.**

**Jan. 21 and 23,** Dr. Pritchard, **Practical Medicine.**

**At 5 p.m.**

**Jan. 20,** Dr. Saunders, **Clinical, II.: with Cases.**

**Jan. 21,** Dr. Ball, **Remarks on Middle Ear Suppuration.**

**Jan. 22,** Mr. Pardoe, **Sterility in the Male.**

**Jan. 23,** Mr. Baldwin, **Practical Surgery, II.**

**Jan. 24,** Mr. Lloyd Williams, **A Discussion of the Dental Conditions which Affect the General Health.**

**NATIONAL HOSPITAL FOR THE PARALYSED AND EPILEPTIC, Queen Square, Bloomsbury, W.C.**

**Lectures at 3.30 p.m.**

**Jan. 21,** Dr. Tooth, **Disseminated Sclerosis.**

**Jan. 24,** Mr. Sargent, **Surgery of the Nervous System.**

**CHARING CROSS HOSPITAL, Endell Street, W.C.**

**Post-Graduate Lecture at 4 p.m.**

**Jan. 23,** Mr. Waterhouse, **Surgical.**

**MOUNT VERNON HOSPITAL POST-GRADUATE COURSE, Fitzroy Square.**

**At 5 p.m.**

**Jan. 23,** Dr. G. F. Johnston, **Pneumococcal Infection.**

**MEDICAL GRADUATES' COLLEGE AND POLYCLINIC, Chenies Street, W.C.**

**Lectures at 5.15 p.m.**

**Jan. 20,** Mr. Lockhart Mummery, **The Symptoms and Diagnosis of Cancer of the Large Intestine.**

**Jan. 21,** Dr. W. Langdon Brown, **What Constitutes Diabetes?**

**Jan. 22,** Dr. A. E. Giles, **Displacement of the Pelvic Organs.**

**Jan. 23,** Dr. G. H. Savage, **Mental Disorders of Childhood.**

**NORTH-EAST LONDON POST-GRADUATE COLLEGE, Prince of Wales' General Hospital, Tottenham, N.**

**Lectures at 4.30 p.m.**

**Jan. 21,** Mr. H. W. Carson, **Diagnosis of Diseases of the Large Intestine.**

**Jan. 23,** Dr. T. R. Whipham, **Pneumonia in Childhood.**

**LONDON SCHOOL OF CLINICAL MEDICINE, Seamen's Hospital, Greenwich.**

**At 2.15 p.m.**

**Jan. 20,** Sir Dyce Duckworth, **Sciatica.**

**At 3.15 p.m.**

**Jan. 23,** Sir Wm. Bennett, **x-Rays in Relation to the Diagnosis of Appendicitis, etc.**

### BUSINESS NOTICES.

**Annual Subscriptions.**

The rate of annual subscriptions to The Hospital, either direct from the Office or through agents, is:—

For the United Kingdom ... ... 15s. a year
To the Colonies and Abroad ... 17s. a year inclusive of postage in each case.

**Subscriptions.**

Subscriptions are payable in advance. Cheques and Postal Orders should be made payable to:—

The Scientific Press, Ltd., and should be crossed: London and County Banking Co., Ltd.

### NOTICES AND ANSWERS TO CORRESPONDENCE.

**Contributions.**

Contributions should be written, or preferably typed, on one side of the paper only, and all articles sent in are accepted upon the distinct understanding that they are forwarded to The Hospital only.

**Correspondence.**

Correspondence on all subjects is invited, but no communication can be entertained if the name and address of the correspondent are not given as a guarantee of good faith, but not necessarily for publication. All correspondents should write on one side of the paper only.

